# RSL3 and Erastin differentially regulate redox signaling to promote Smac mimetic-induced cell death

**DOI:** 10.18632/oncotarget.11687

**Published:** 2016-08-29

**Authors:** Jasmin Dächert, Hannah Schoeneberger, Katharina Rohde, Simone Fulda

**Affiliations:** ^1^ Institute for Experimental Cancer Research in Pediatrics, Goethe-University, Frankfurt, Germany; ^2^ German Cancer Consortium (DKTK), Heidelberg, Germany; ^3^ German Cancer Research Center (DKFZ), Heidelberg, Germany

**Keywords:** Smac mimetic, cell death, ferroptosis, redox, ROS

## Abstract

Redox mechanisms play an important role in the control of various signaling pathways. Here, we report that Second mitochondrial activator of caspases (Smac) mimetic-induced cell death is regulated by redox signaling. We show that RSL3, a glutathione (GSH) peroxidase (GPX) 4 inhibitor, or Erastin, an inhibitor of the cystine/glutamate antiporter, cooperate with the Smac mimetic BV6 to induce reactive oxygen species (ROS)-dependent cell death in acute lymphoblastic leukemia (ALL) cells. Addition of the caspase inhibitor N-benzyloxycarbonyl-Val-Ala-Asp-fluoromethylketone (zVAD.fmk) fails to rescue ROS-induced cell death, demonstrating that RSL3/BV6- or Erastin/BV6-induced cell death occurs in a caspase-independent manner. Interestingly, the iron chelator Deferoxamine (DFO) significantly inhibits RSL3/BV6-induced cell death, whereas it is unable to rescue cell death by Erastin/BV6, showing that RSL3/BV6-, but not Erastin/BV6-mediated cell death depends on iron. ROS production is required for both RSL3/BV6- and Erastin/BV6-induced cell death, since the ROS scavenger α-tocopherol (α-Toc) rescues RSL3/BV6- and Erastin/BV6-induced cell death. By comparison, genetic or pharmacological inhibition of lipid peroxidation by GPX4 overexpression or ferrostatin (Fer)-1 significantly decreases RSL3/BV6-, but not Erastin/BV6-induced cell death, despite inhibition of lipid peroxidation upon exposure to RSL3/BV6 or Erastin/BV6. Of note, inhibition of lipid peroxidation by Fer-1 protects from RSL3/BV6-, but not from Erastin/BV6-stimulated ROS production, indicating that other forms of ROS besides lipophilic ROS occur during Erastin/BV6-induced cell death. Taken together, RSL3/BV6 and Erastin/BV6 differentially regulate redox signaling and cell death in ALL cells. While RSL3/BV6 cotreatment induces ferroptotic cell death, Erastin/BV6 stimulates oxidative cell death independently of iron. These findings have important implications for the therapeutic targeting of redox signaling to enhance Smac mimetic-induced cell death in ALL.

## INTRODUCTION

Evasion of programmed cell death is one of the hallmarks of cancer [1] and contributes to tumor formation and treatment failure [2]. The efficacy of anticancer therapies critically depends on their ability to engage cancer cell death, highlighting the need to develop new concepts to trigger programmed cell death in cancer cells. The redox state of a cell plays an important role in the control of various cellular processes, including cell death pathways, and maintenance of redox homeostasis is critical for cell survival [3]. As the accumulation of ROS can be detrimental to the cell, several antioxidant defense systems exist for their detoxification, for example GSH, the most abundant non-protein thiol [3]. Accumulation of ROS in the vicinity of lipid membranes can trigger lipid peroxidation, which can lead to cell death via the destruction of membranes [4].

The membrane redox state is governed on the one side by the generation of lipid peroxides and on the other side by membrane-associated enzymatic and non-enzymatic peroxide scavengers. Within the family members of GPX that catalyze the reduction of hydrogen and lipid peroxides, only GPX4 has been described to directly detoxify lipid hydroperoxides within biological membranes [5]. In addition, non-enzymatic peroxide scavengers can neutralize membrane lipidperoxyl radicals, for example Fer-1, described as a small-molecule inhibitor of lipid peroxidation [6], and lipophilic antioxidants such as α-Toc [7]. Disturbance of the membrane redox state can lead to permeabilization of membranes and subsequently to cell death. Several ROS-generating enzymes contain iron or iron derivatives as essential co-factors for their proper function, for example lipoxygenases (LOX), nicotinamide adenine dinucleotide phosphate hydride (NADPH) oxidases (NOX), xanthine oxidase, and cytochrome P450 enzymes [8]. In addition, redox-active labile iron pools can directly catalyze free radical formation via Fenton chemistry [9].

There are different modes of programmed cell death [10]. Among them, ferroptosis is a newly defined form of regulated, oxidative cell death that is characterized by lipid peroxidation, generation of lipid-based ROS and its dependency on iron [11]. As ferroptosis represents a form of non-apoptotic cell death, therapeutic induction of ferroptosis has recently emerged as a new strategy to trigger cancer cell death. Ferroptosis-inducing compounds have been categorized into two classes based on their mode of GPX4 inhibition [12]. One class, including Erastin, indirectly inhibits GPX4 through GSH depletion, as GSH is an essential cofactor of GPXs. The second class, e.g. RSL3, directly inhibits GPX4 without GSH depletion [12]. In the present study, we investigated the question whether prototypic ferroptosis-inducing compounds such as RSL3 and Erastin modulate Smac mimetic-induced cell death in ALL, the most frequent childhood malignancy [13].

## RESULTS

### BV6 cooperates with RSL3 or Erastin to induce cell death, accompanied by ROS production and lipid peroxidation

**Figure 1:**
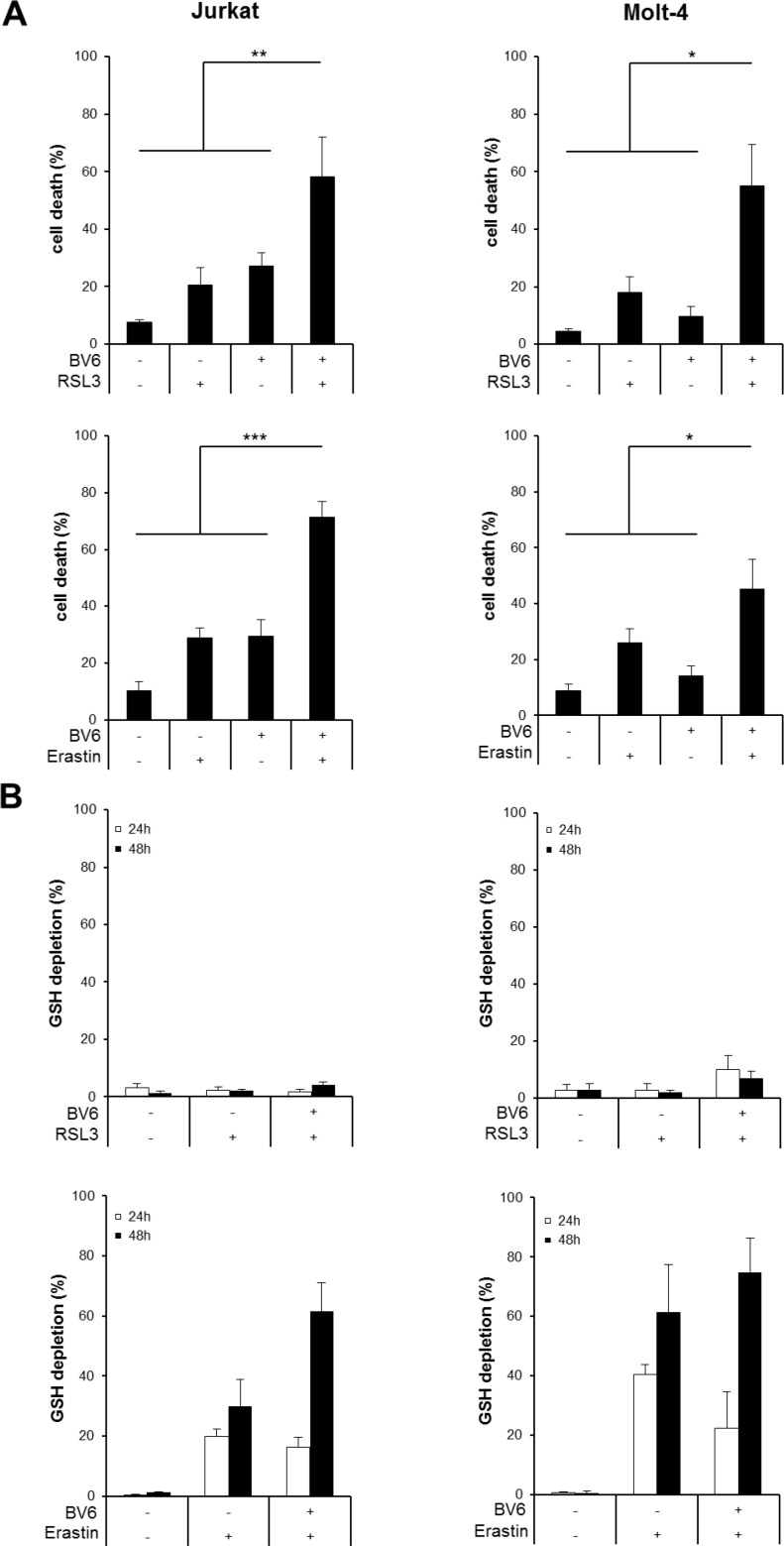

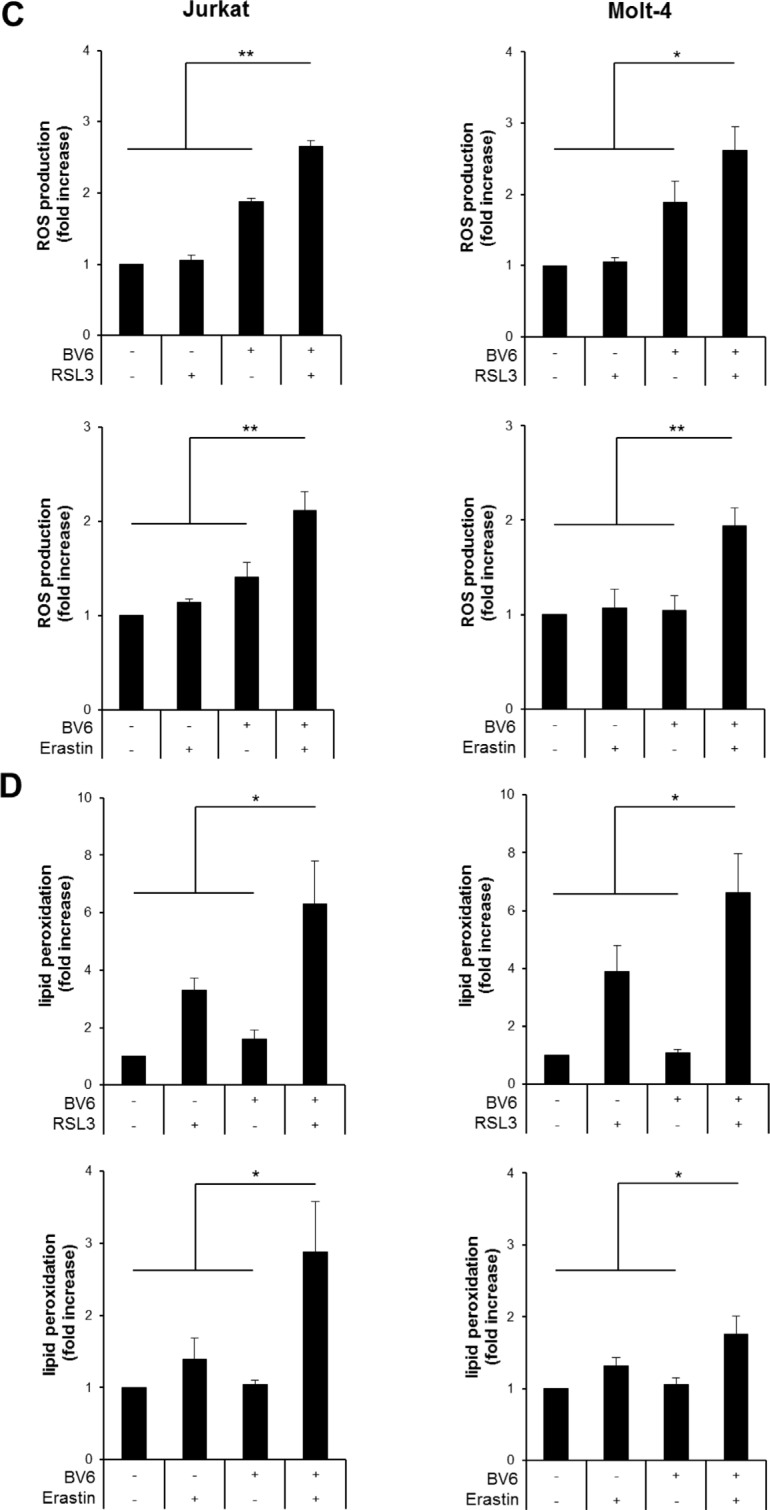
BV6 cooperates with RSL3 or Erastin to induce cell death, accompanied by ROS production and lipid peroxidation (**A**) ALL cells were treated for 24 hours (upper panels) or 48 hours (lower panels) with BV6 (Jurkat: 5 μM, Molt-4: 4 μM), RSL3 (Jurkat: 0.1 μM; Molt-4: RSL3 0.075 μM) and/or Erastin (Jurkat: 5 μM; Molt-4: 7.5 μM). Cell death was determined by FSC/SSC analysis and flow cytometry. B-D, ALL cells were treated with BV6 (Jurkat: 7.5 μM, Molt-4: 5 μM), RSL3 (Jurkat: 0.1 μM, Molt-4: 0.3 μM) and/or Erastin (Jurkat: 5 μM, Molt-4: 15 μM). GSH levels were measured after 24 or 48 hours in PI-negative cells using the fluorescent dye Monobromobimane and flow cytometry and the percentage of GSH depletion is shown (**B**). ROS production was determined after 15 hours (Jurkat) or 24 hours (Molt-4) by flow cytometry in PI-negative cells using the fluorescent dye CellROX and is shown as fold increase (**C**). Lipid peroxidation was assessed after 15 hours (Jurkat) or 24 hours (Molt-4) by flow cytometry in PI-negative cells using the fluorescent dye BODIPY-C11 and is shown as fold increase (**D**). Mean and SD of at least three experiments performed in triplicate are shown; *P < 0.05; **P < 0.01; ***P < 0.001.

To explore whether prototypic ferroptosis-inducing compounds such as RSL3 and Erastin modulate Smac mimetic-induced cell death in ALL cells, we tested the antileukemic activity of the bivalent Smac mimetic BV6 that antagonizes cellular Inhibitor of Apoptosis (cIAP) and x-linked Inhibitor of Apoptosis (XIAP) proteins [14] together with RSL3 and Erastin that were recently described as two prototypic ferroptosis-inducing compounds [12]. RSL3 has been identified as an inhibitor of GPX4 [12], the only GPX family member that can directly reduce lipid hydroperoxides within biological membranes [5]. Erastin blocks the cystine/glutamate antiporter at the plasma membrane that provides cystine for the synthesis of the antioxidant tripeptide GSH, which results in the depletion of cellular GSH levels [15]. Of note, BV6 cooperated with RSL3 to significantly increase cell death compared to treatment with either agent alone in two ALL cell lines (Figure 1A). Similarly, BV6 acted in concert with Erastin to trigger cell death in ALL cells (Figure 1A).

To explore the underlying mechanisms of the observed cooperative induction of cell death by the cotreatment with BV6 and RSL3 or Erastin, we determined cellular GSH levels. Erastin alone and in combination with BV6 caused a significant reduction of GSH levels, consistent with the described mode of action of Erastin as an inhibitor of the cystine/glutamate antiporter. In contrast, GSH levels remained largely unaffected upon treatment with BV6 [16] or RSL3 (Figure 1B), in line with the reported function of RSL3 as GPX4 inhibitor that triggers ferroptosis without changes in GSH levels [12].

Since inhibition of GPX4 or depletion of cellular GSH levels facilitate the accumulation of ROS [3], we hypothesized that RSL3 or Erastin act together with BV6 to cause alterations in ROS levels. To clarify this hypothesis, we analyzed cellular ROS levels in ALL cells before they succumb to cell death using the fluorescent ROS-sensitive dye CellROX. Importantly, both RSL3 and Erastin cooperated with BV6 to significantly increase ROS levels compared to either treatment alone (Figure 1C).

GPX4 inactivation has been shown to cause accumulation of lipid peroxides [12], and ROS accumulation in the vicinity of biomembranes has been described to favor lipid peroxidation [4]. Therefore, we next analyzed lipid peroxidation by staining viable (i.e. propidium iodide (PI)-negative) cells with the membrane-targeted lipid ROS sensor BODIPY-C11, a fluorescent dye that detects lipid peroxides [17]. Of note, RSL3 and Erastin acted together with BV6 to significantly enhance lipid peroxidation compared to treatment with either agent alone (Figure 1D).

Collectively, these findings demonstrate that BV6 cooperates with RSL3 or Erastin to induce cell death, which is accompanied by accumulation of ROS and lipid peroxidation.

### RSL3/BV6 or Erastin/BV6 cotreatment triggers caspase-independent lipid peroxidation-induced cell death

**Figure 2:**
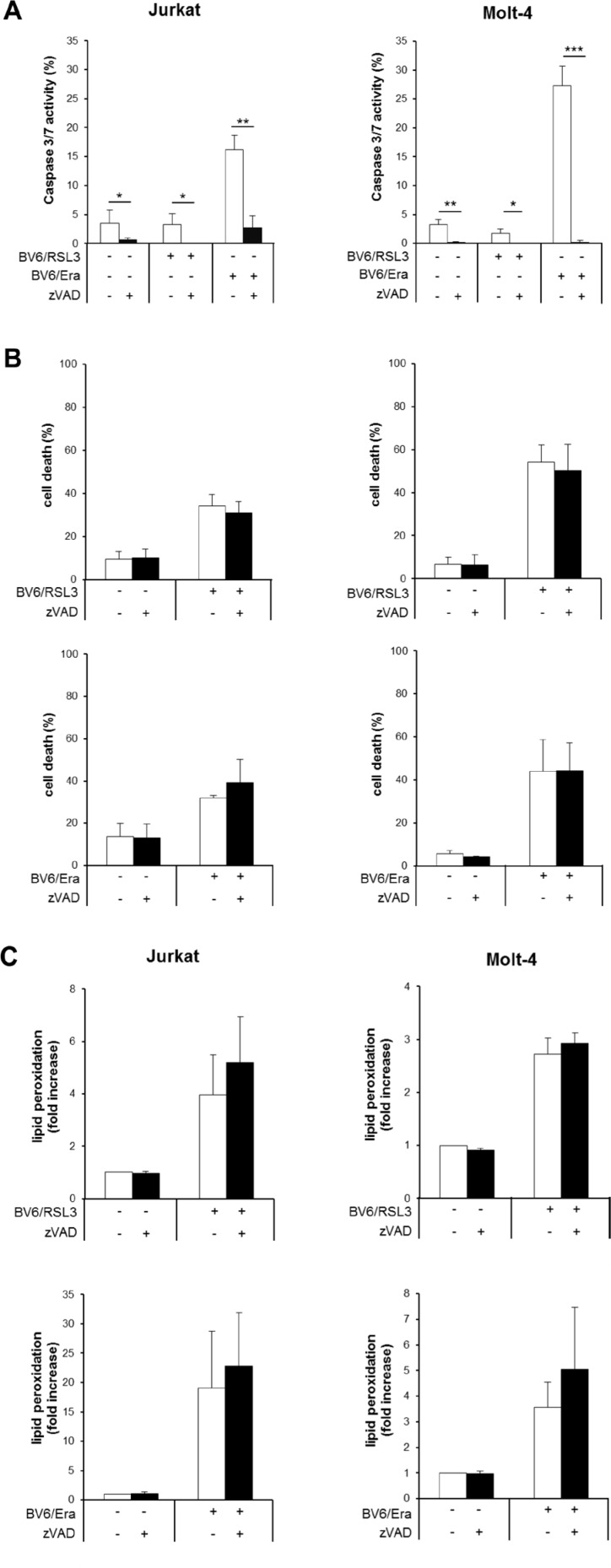
RSL3/BV6- or Erastin/BV6- cotreatment triggers caspase-independent lipid peroxidation-induced cell death (**A**–**C**) ALL cells were treated with BV6 (Jurkat: 5 μM, Molt-4: 4 μM), RSL3 (Jurkat: 0.1 μM; Molt-4: RSL3 0.075 μM) and/or Erastin (Era) (Jurkat: 5 μM; Molt-4: 7.5 μM) in the presence or absence of 20 μM zVAD.fmk, which was added 2 hours before treatment. Caspase-3/7 activity was determined after 48 hours by Cell Event Caspase-3/7 Green Detection Reagent and ImageXpress Micro XLS system (A). Cell death was determined after 12 hours (Jurkat cells: Erastin/BV6) or 24 hours (Jurkat cells: RSL3/BV6, Molt-4 cells: RSL3/BV6, Erastin/BV6) by FSC/SSC analysis and flow cytometry (B). Lipid peroxidation was assessed after 18 hours (Erastin/BV6) or 24 hours (RSL3/BV6) by flow cytometry in PI-negative cells using the fluorescent dye BODIPY-C11 and is shown as fold increase compared to untreated cells (C). Mean and SD of at least three experiments performed in triplicate are shown; *P < 0.05; **P < 0.01; ***P < 0.001.

Next, we explored the involvement of caspases by monitoring caspase-3/7 activity. While cotreatment with RSL3/BV6 did not alter caspase-3/7 activity compared to untreated control cells, Erastin/BV6 caused a significant increase in caspase-3/7 activity (Figure 2A). To explore whether caspase activation is necessary for the induction of cell death, we used the pan-caspase inhibitor zVAD.fmk. Of note, the addition of zVAD.fmk failed to protect ALL cells from RSL3/BV6- or Erastin/BV6- stimulated cell death (Figure 2B). Control experiments showed that zVAD.fmk prevented Erastin/BV6-stimulated caspase-3/7 activation as well as low constitutive caspase-3/7 activity (Figure 2A), confirming that zVAD.fmk indeed blocks caspase activity in this setting. In addition, zVAD.fmk was unable to prevent RSL3/BV6- or Erastin/BV6-induced lipid peroxidation (Figure 2C). These findings demonstrate that RSL3/BV6 or Erastin/BV6 induce cell death in a caspase-independent lipid peroxidation manner.

### RSL3/BV6 but not Erastin/BV6 cotreatment triggers iron-dependent cell death

**Figure 3:**
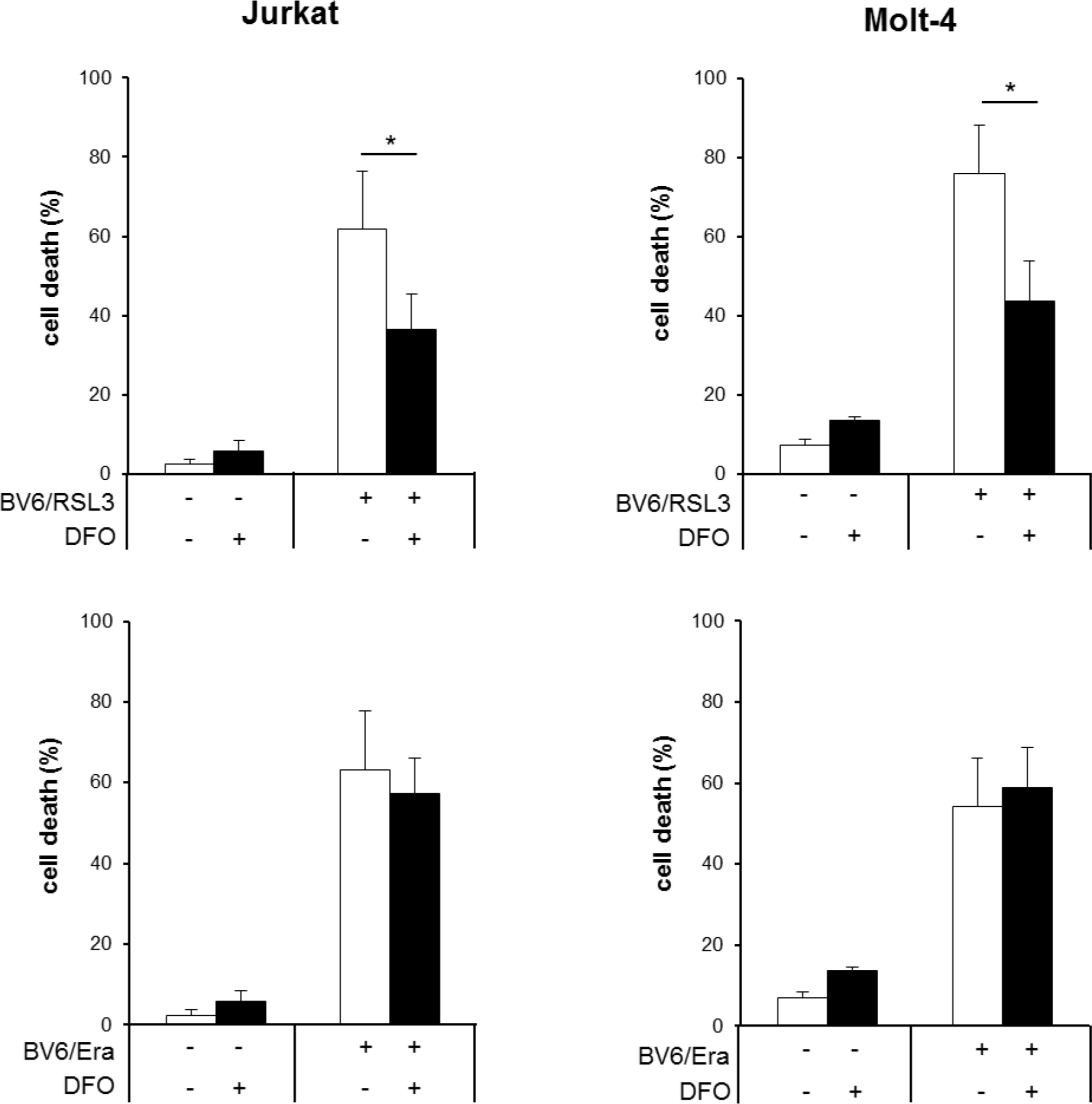
RSL3/BV6 but not Erastin/BV6 cotreatment triggers iron-dependent cell death ALL cells were treated for 24 hours with BV6 (Jurkat: 5 μM, Molt-4: 4 μM), RSL3 (Jurkat: 0.1 μM; Molt-4: RSL3 0.075 μM) and/or Erastin (Era) (Jurkat: 5 μM; Molt-4: 7.5 μM) in the presence or absence of 25 μM DFO, which was added 2 hours before treatment. Cell death was determined by FSC/SSC analysis and flow cytometry. Mean and SD of at least three experiments performed in triplicate are shown; *P < 0.05.

To explore the involvement of ferroptotic cell death, which is characterized by its dependency on iron, we tested the effect of the iron chelator DFO. Importantly, DFO significantly reduced RSL3/BV6-stimulated cell death, whereas it was unable to block Erastin/BV6-induced cell death even at higher DFO concentrations (Figure 3, Supplementary Figure S1A). Treatment with a high concentration of RSL3 was used as a positive control for ferroptosis (Supplementary Figure S1B). This indicates that RSL3/BV6 but not Erastin/BV6 cotreatment triggers ferroptotic cell death. To confirm that the effects brought about by Erastin are due to cystine deprivation leading to GSH depletion, we analyzed the effects of the thiol-containing compound N-acetylcysteine (NAC). The presence of NAC, which almost completely suppressed ROS production, significantly decreased BV6/Erastin-induced cell death (Supplementary Figure S2).

### Erastin/BV6/zVAD.fmk cotreatment triggers Receptor-interacting protein (RIP)1- and RIP3-independent cell death

**Figure 4:**
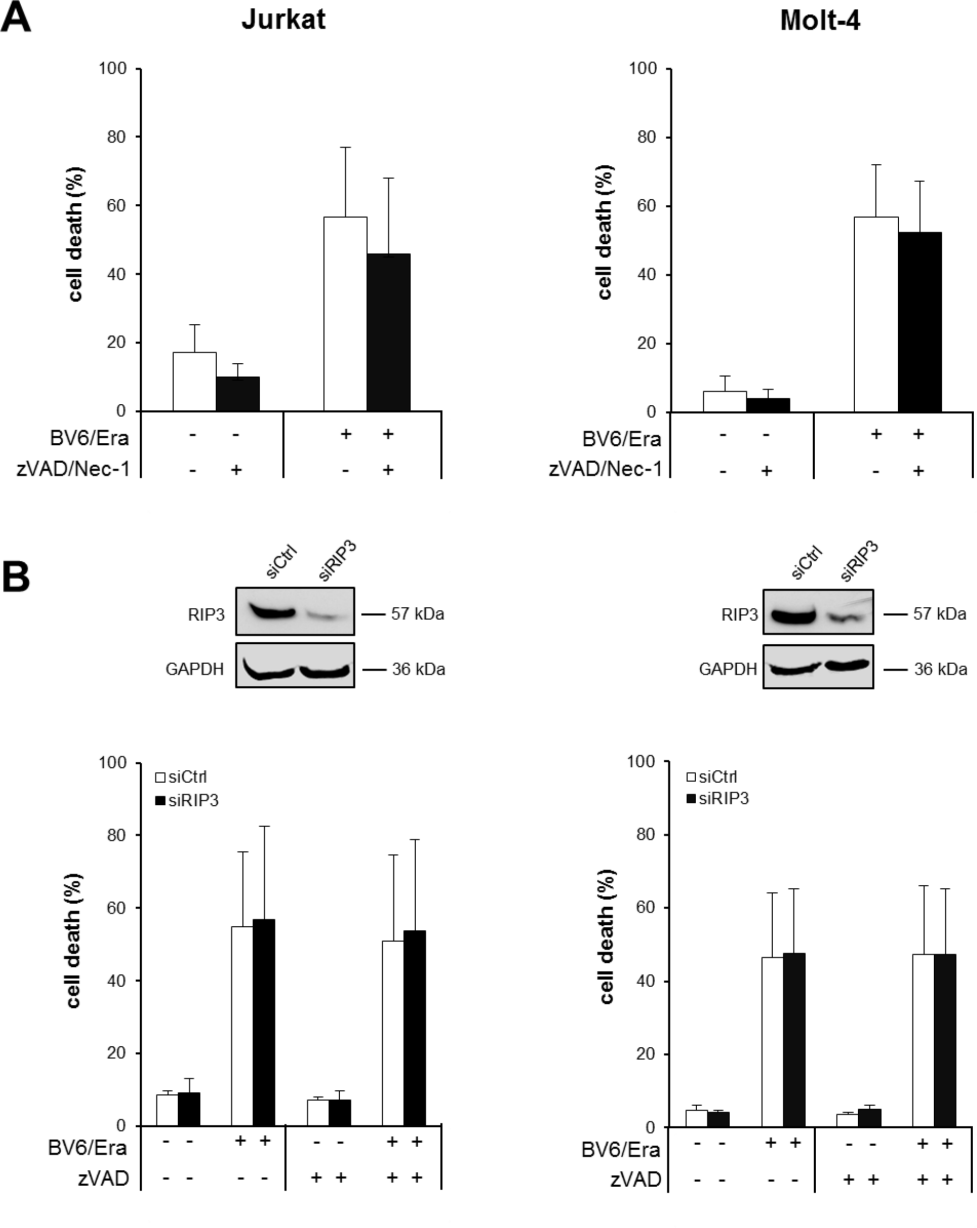
Erastin/BV6/zVAD.fmk cotreatment triggers RIP1- and RIP3-independent cell death (**A**) ALL cells were treated for 24 hours with BV6 (Jurkat: 5 μM, Molt-4: 4 μM) and/or Erastin (Era) (Jurkat: 5 μM; Molt-4: 7.5 μM) in the presence or absence of 20 μM zVAD.fmk and 15 μM Nec-1, which were added 2 hours before treatment. Cell death was determined by FSC/SSC analysis and flow cytometry. Mean and SD of at least three experiments performed in triplicate are shown. (**B**) ALL cells were transiently transfected with siRNA against RIP3 or control siRNA and RIP3 protein expression was determined by Western blotting (upper panels). Cells were treated for 24 hours with BV6 (Jurkat: 5 μM, Molt-4: 4 μM) and/or Erastin (Jurkat: 5 μM; Molt-4: 7.5 μM) in the presence or absence of 20 μM zVAD.fmk, which was added 2 hours before treatment and cell death was determined by FSC/SSC analysis and flow cytometry (lower panels). Mean and SD of at least three experiments performed in triplicate are shown.

Necroptosis represents another mode of programmed cell death that can be engaged in particular upon caspase inhibition [18]. To test whether Erastin/BV6 cotreatment triggers necroptosis when caspase activation is inhibited by zVAD.fmk we used pharmacological and genetic approaches to block key components of necroptosis signaling. Notably, the addition of the RIP1 kinase inhibitor necrostatin-1 (Nec-1) failed to prevent Erastin/BV6/zVAD.fmk-induced cell death (Figure 4A), whereas Nec-1 significantly reduced cell death upon treatment with tumor necrosis factor (TNF)α/BV6/zVAD.fmk that was used as a positive control for necroptotic cell death (Supplementary Figure S3A). Similarly, knockdown of RIP3 did not alter Erastin/BV6-driven cell death in the presence or absence of zVAD.fmk (Figure 4B), whereas TNFα/BV6/zVAD.fmk-stimulated cell death was significantly decreased in RIP3 knockdown cells (Supplementary Figure S3B). This set of experiments shows that RIP1 kinase activity and RIP3 are dispensable for Erastin/BV6-mediated cell death, suggesting that Erastin/BV6 cotreatment does not induce necroptosis when caspase activation is inhibited.

### α-Toc inhibits RSL3/BV6- or Erastin/BV6-induced lipid peroxidation and cell death

**Figure 5:**
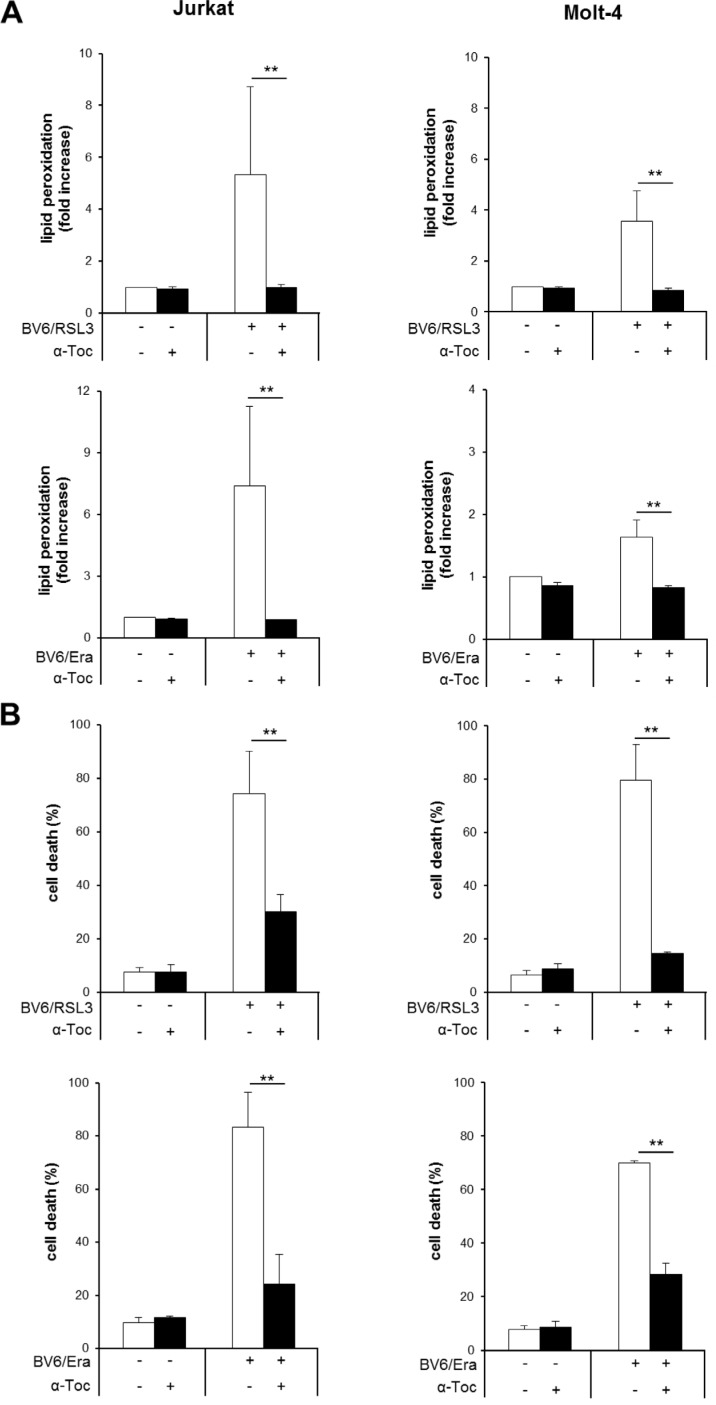
α-Toc inhibits RSL3/BV6- or Erastin/BV6-induced lipid peroxidation and cell death ALL cells were treated with BV6 (Jurkat: 7.5 μM, Molt-4: 5 μM), RSL3 (Jurkat: 0.1 μM, Molt-4: 0.3 μM) and/or Erastin (Era) (Jurkat: 5 μM, Molt-4: 15 μM) in the presence or absence of 100 μM α-Toc. Lipid peroxidation was assessed after 15 hours (Jurkat: RSL3/BV6) or 24 hours (Jurkat: Erastin/BV6, Molt-4: RSL3/BV6, Erastin/BV6) by flow cytometry in PI-negative cells using the fluorescent dye BODIPY-C11 and is shown as fold increase (**A**). Cell death was determined by FSC/SSC analysis and flow cytometry (**B**). Mean and SD of three experiments performed in triplicate are shown; **P < 0.01.

To investigate the contribution of ROS to cell death we used α-Toc, a lipophilic ROS scavenger. The addition of α-Toc significantly reduced lipid peroxidation upon cotreatment with RSL3/BV6 or Erastin/BV6 in several ALL cell lines (Figure 5A, Supplementary Figure S4A). Importantly, α-Toc significantly rescued both Erastin/BV6- and RSL3/BV6-stimulated cell death in several ALL cell lines (Figure 5B, Supplementary Figure S4B). This indicates that inhibition of ROS accumulation protects cells from RSL3/BV6- or Erastin/BV6-induced lipid peroxidation and cell death.

### GPX4 overexpression inhibits RSL3/BV6- but not Erastin/BV6-induced cell death, while it prevents both RSL3/BV6- and Erastin/BV6-induced lipid peroxidation

**Figure 6:**
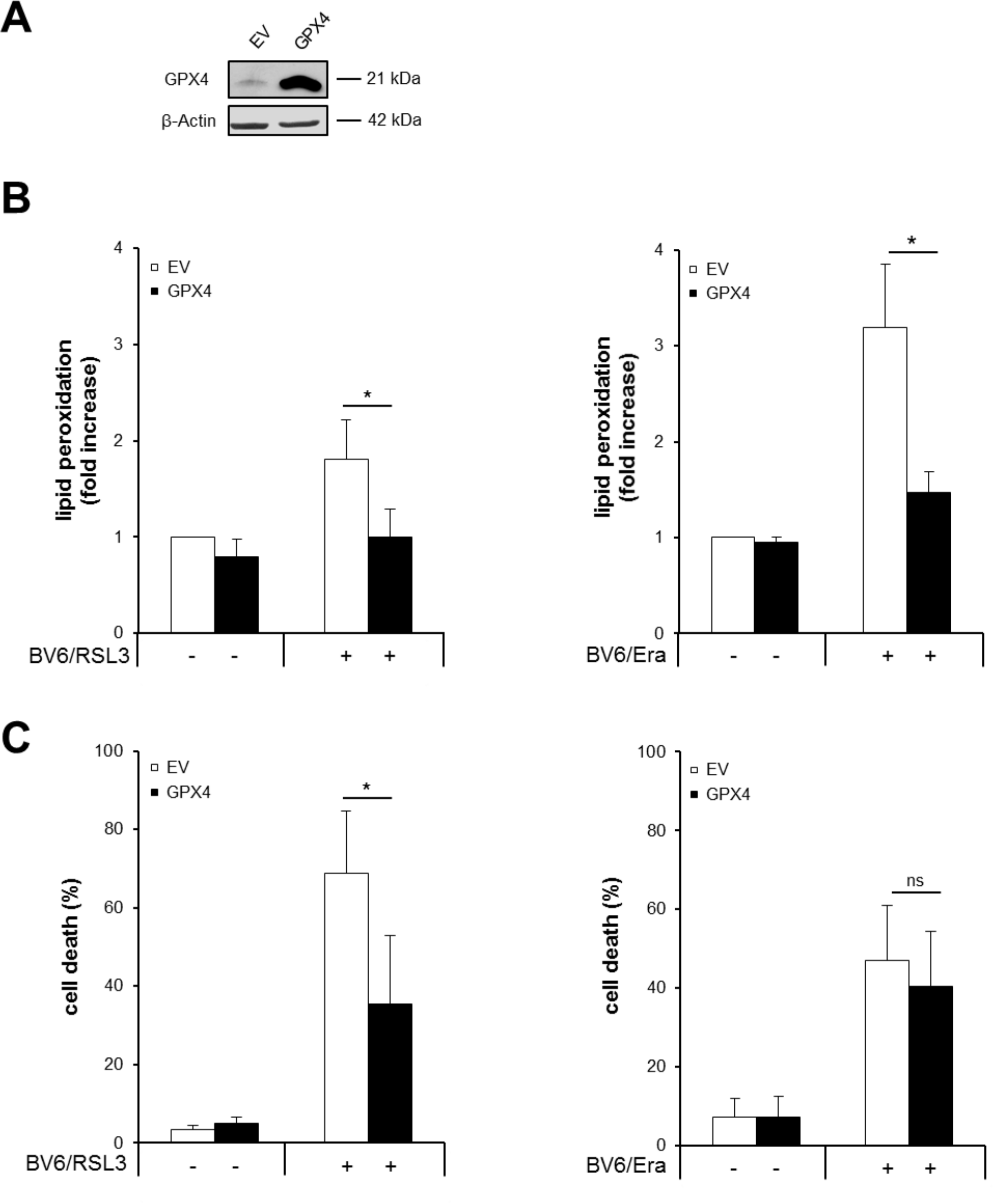
GPX4 overexpression inhibits RSL3/BV6- but not Erastin/BV6-induced cell death, while it prevents both RSL3/BV6- and Erastin/BV6-induced lipid peroxidation Molt-4 cells were stably transduced with empty vector (EV) or a vector containing GPX4. (**A**) Protein expression of GPX4 was analyzed by Western blotting. (**B** and **C**) Cells were treated with 4 μM BV6, 0.075 μM RSL3 and/or 7.5 μM Erastin (Era). Lipid peroxidation was assessed after 18 hours (Erastin/BV6) or 24 hours (RSL3/BV6) by flow cytometry in PI-negative cells using the fluorescent dye BODIPY-C11 and is shown as fold increase (B). Cell death was determined after 24 hours by FSC/SSC analysis and flow cytometry (C). Mean and SD of at least three experiments performed in triplicate are shown; *P < 0.05, ns, not significant.

To clarify the role of lipid peroxidation in RSL3/BV6- or Erastin/BV6-induced cell death we ectopically expressed GPX4, which is described to protect biomembranes from oxidative stress by specifically reducing lipid peroxides [5]. Ectopic expression of GPX4 in stably transfected Molt-4 cells was confirmed by Western blotting (Figure 6A). GPX4 overexpression significantly reduced RSL3/BV6- or Erastin/BV6-induced lipid peroxidation (Figure 6B), consistent with the reported function of GPX4 to scavenge lipid peroxides [5]. Also, overexpression of GPX4 significantly decreased RSL3/BV6-stimulated cell death, whereas it failed to rescue Erastin/BV6-mediated cell death (Figure 6C). These findings demonstrate that GPX4 overexpression protects ALL cells from RSL3/BV6- but not Erastin/BV6-induced cell death, while it prevents both RSL3/BV6- and Erastin/BV6-induced lipid peroxidation.

### Fer-1 inhibits RSL3/BV6- but not Erastin/BV6-induced cell death, while it prevents both RSL3/BV6- and Erastin/BV6-induced lipid peroxidation

**Figure 7:**
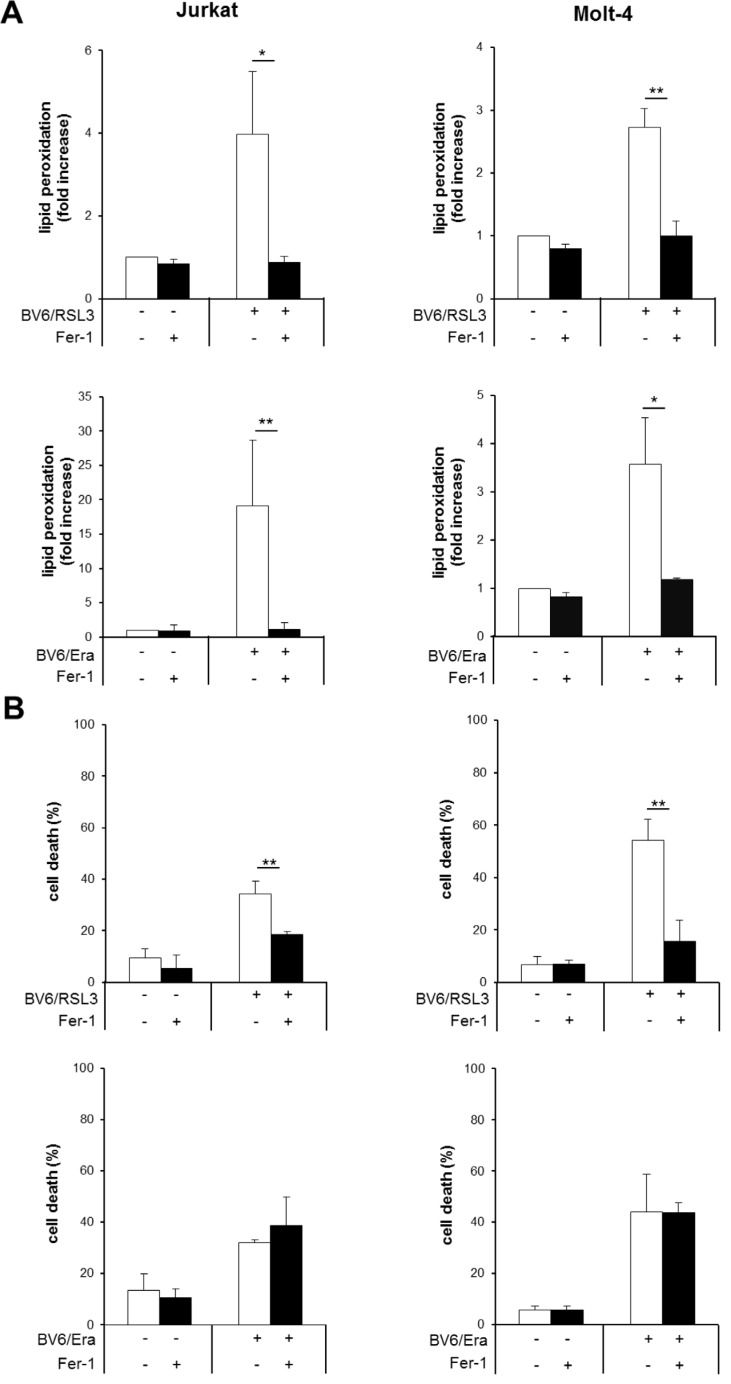
Fer-1 inhibits RSL3/BV6- but not Erastin/BV6-induced cell death, while it prevents both RSL3/BV6- and Erastin/BV6-induced lipid peroxidation ALL cells were treated with BV6 (Jurkat: 5 μM, Molt-4: 4 μM), RSL3 (Jurkat: 0.1 μM; Molt-4: RSL3 0.075 μM) and/or Erastin (Era) (Jurkat: 5 μM; Molt-4: 7.5 μM) in the presence or absence of 5 μM Fer-1, which was added 2 hours before treatment. Lipid peroxidation was assessed after 12 hours (Jurkat: Erastin/BV6), 18 hours (Molt-4: Erastin/BV6) or 24 hours (Jurkat: RSL3/BV6, Molt-4: RSL3/BV6) by flow cytometry in PI-negative cells using the fluorescent dye BODIPY-C11 and is shown as fold increase (**A**). Cell death was determined by FSC/SSC analysis and flow cytometry (**B**). Mean and SD of three experiments performed in triplicate are shown; *P < 0.05; **P < 0.01.

In addition to genetic blockage of lipid peroxidation by GPX4 overexpression, we also employed a pharmacological approach to further investigate the role of lipid peroxidation. To this end, we used Fer-1, described as a small-molecule inhibitor of lipid peroxidation [6]. We found that Fer-1 significantly reduced both RSL3/BV6-and Erastin/BV6-mediated lipid peroxidation in both ALL cell lines (Figure 7A, Supplementary Figure S5A), in line with its reported mode of action [6]. Of note, Fer-1 significantly decreased RSL3/BV6-stimulated cell death, whereas it failed to prevent Erastin/BV6-induced cell death (Figure 7B). These findings are consistent with our results obtained by GPX4 overexpression and emphasize that inhibition of lipid peroxidation is not sufficient to protect from Erastin/BV6-induced cell death.

### α-Toc rescues both RSL3/BV6- and Erastin/BV6-induced ROS production, whereas Fer-1 prevents RSL3/BV6- but not Erastin/BV6-stimulated ROS generation

**Figure 8:**
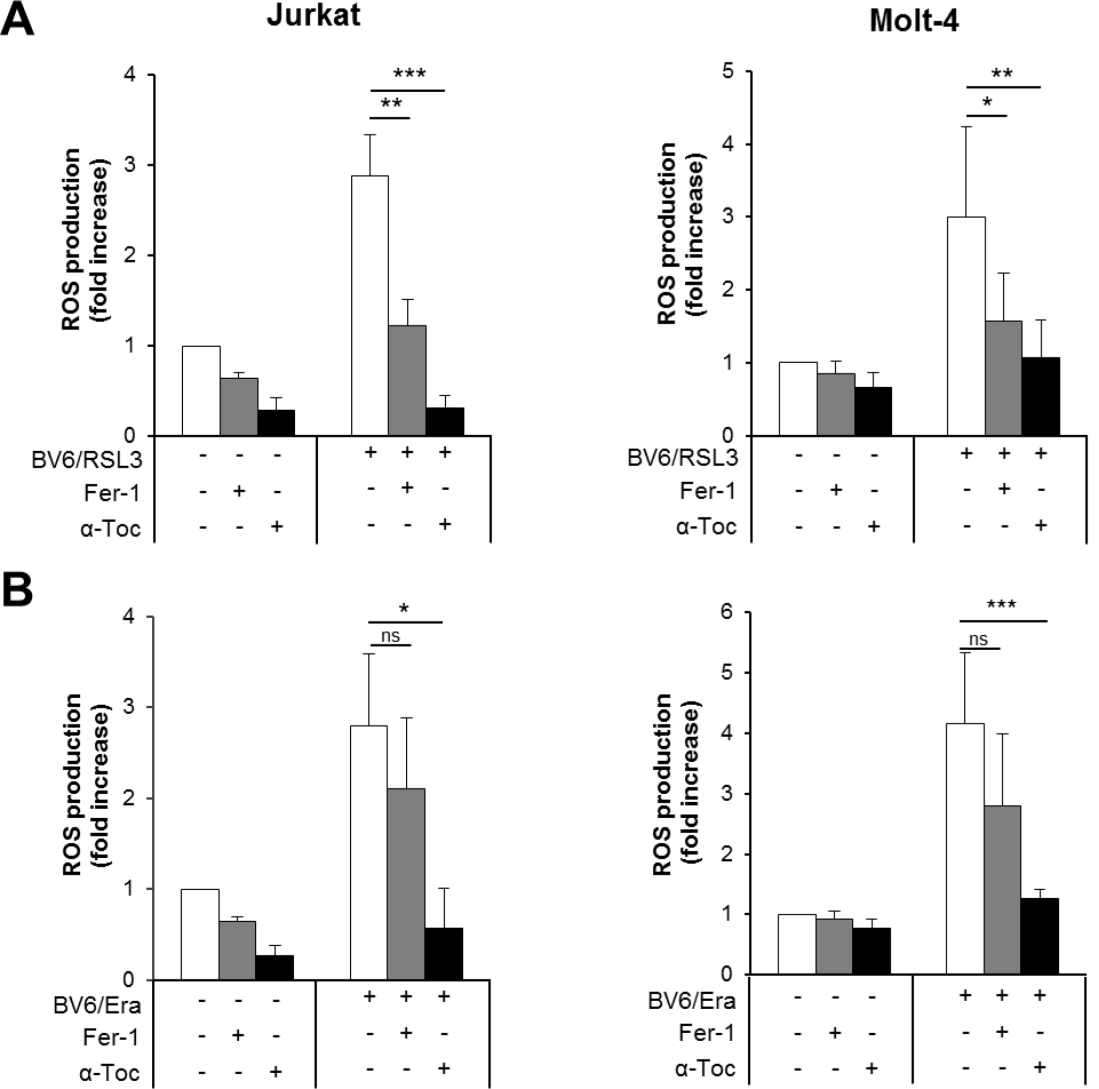
α-Toc rescues both RSL3/BV6- and Erastin/BV6-induced ROS production, whereas Fer-1 prevents RSL3/BV6- but not Erastin/BV6-stimulated ROS generation ALL cells were treated with BV6 (Jurkat: 5 μM, Molt-4: 4 μM), RSL3 (Jurkat: 0.1 μM; Molt-4: RSL3 0.075 μM) and/or Erastin (Era) (Jurkat: 5 μM; Molt-4: 7.5 μM) in the presence or absence of 100 μM α-Toc or 5 μM Fer-1, which were added 2 hours before treatment. ROS production was determined after 15 hours by flow cytometry in PI-negative cells using the fluorescent dye CellROX and is shown as fold increase with mean and SD of three experiments performed in triplicate; *P < 0.05; **P < 0.01; ***P < 0.001.

Our results obtained so far demonstrate that inhibition of ROS accumulation by α-Toc rescues both RSL3/BV6- and Erastin/BV6-induced cell death, while genetic or pharmacological inhibition of lipid peroxidation protect cells from RSL3/BV6- but not Erastin/BV6-stimulated cell death. Therefore, we hypothesized that α-Toc and Fer-1 have a differential ability to block ROS accumulation upon cotreatment with RSL3/BV6 or Erastin/BV6. To test this hypothesis we analyzed the effect of α-Toc and Fer-1 on ROS accumulation in response to RSL3/BV6 or Erastin/BV6 treatment. Importantly, α-Toc significantly reduced both RSL3/BV6- and Erastin/BV6-stimulated ROS production (Figure 8A, Supplementary Figure S5B). In contrast, Fer-1 significantly decreased RSL3/BV6- but not Erastin/BV6-mediated ROS accumulation (Figure 8B). These findings underline that mainly lipophilic ROS are produced upon RSL3/BV6 cotreatment, since inhibition of lipid peroxidation can prevent overall ROS accumulation. In contrast, other sources of toxic ROS besides lipophilic ROS are involved in Erastin/BV6-induced cell death.

## DISCUSSION

Redox signaling plays an important role in the control of cell death pathways. However, the mechanisms of action are yet poorly understood. Here, we report that Smac mimetic-induced cell death is regulated by redox signaling. We show that the GPX4 inhibitor RSL3 or Erastin, an inhibitor of the cystine/glutamate antiporter, cooperate with the Smac mimetic BV6 to induce ROS-dependent cell death in ALL cells. Importantly, RSL3/BV6 cotreatment induces ferroptotic cell death, whereas Erastin/BV6 cotreatment stimulates oxidative cell death independently of iron. These conclusions are drawn on the basis of the following pieces of experimental evidence. First, RSL3/BV6 or Erastin/BV6 trigger caspase-independent lipid peroxidation-induced cell death, since the caspase inhibitor zVAD.fmk fails to rescue lipid peroxidation and cell death. Second, the iron chelator DFO significantly inhibits RSL3/BV6- but not Erastin/BV6-induced cell death, demonstrating that RSL3/BV6 triggers an iron-dependent form of cell death, whereas Erastin/BV6-induced cell death occurs independently of iron. Third, ROS production is required for both RSL3/BV6- and Erastin/BV6-induced cell death, since inhibition of ROS accumulation by α-Toc rescues RSL3/BV6- as well as Erastin/BV6-induced cell death. Fourth, genetic or pharmacological inhibition of lipid peroxidation by GPX4 overexpression or Fer-1 significantly decreases RSL3/BV6- but not Erastin/BV6-induced cell death, despite inhibition of lipid peroxidation upon exposure to RSL3/BV6 or Erastin/BV6, indicating that lipid peroxidation is critically involved in RSL3/BV6-induced cell death. Fifth, we demonstrate that ROS production mainly triggers lipid peroxidation during RSL3/BV6-induced cell death, since pharmacological inhibition of lipid peroxidation by Fer-1 significantly protects from ROS production and since the lipophilic ROS scavenger α-Toc reduces ROS production to a similar extent. In contrast, Fer-1 fails to prevent Erastin/BV6-induced ROS production, while α-Toc protects from Erastin/BV6-stimulated lipid peroxidation, indicating that, besides lipophilic ROS production, also other forms of ROS are generated during Erastin/BV6-induced cell death. Taken together, RSL3/BV6 and Erastin/BV6 differentially regulate redox signaling and cell death in ALL cells. While RSL3/BV6 cotreatment induces ferroptosis, as demonstrated by four characteristic features of ferroptotic cell death, that is dependency on iron, lipid peroxidation and ROS production without the requirement of caspases, Erastin/BV6 cotreatment triggers oxidative cell death in an iron-independent lipid peroxidation manner.

While both RSL3 and Erastin have been classified as prototypic ferroptosis-inducing compounds when used as single agents at cytotoxic concentrations that trigger cell death [12], in the present study we show that RSL3 but not Erastin induces ferroptosis when used at subcytotoxic doses in combination with the Smac mimetic BV6. This points to the existence of differences in the signaling pathways that are engaged upon cotreatment with RSL3/BV6 *versus* Erastin/BV6, which could be explained by the different modes of action of RSL3 and Erastin. RSL3 has been characterized as a small-molecule GPX4 inhibitor [12]. According to the current model, inhibition of GPX4 leads to the accumulation of lipid peroxides which, via an iron-catalyzed reaction, generate toxic lipid radicals that are lethal to the cell [19]. Erastin depletes GSH by inhibiting the cystine/glutamate antiporter that provides cystine for GSH synthesis [15]. GSH depletion not only indirectly impairs GPX4 function that requires GSH as an essential cofactor [5], but also reduces the antioxidant capacity of the cell, since GSH, being the most abundant non-protein thiol, is one of the key antioxidant defense systems of the cell [3], thereby favoring ROS accumulation. Accumulation of ROS can be detrimental to the cell via various mechanisms. In addition, Erastin has been reported to inhibit voltage-dependent anion-selective channel protein (VDAC)2/3, a component of the mitochondrial permeability transition pore [20]. Thus, while both RSL3 and Erastin act in concert with the Smac mimetic BV6 to stimulate ROS-dependent cell death in ALL cells, there are differences in the underlying molecular mechanisms and the resulting type of cell death.

Since inhibition of caspases has been reported to favor a switch from apoptotic to necroptotic cell death, we tested the hypothesis according to which Erastin/BV6 cotreatment induces necroptosis when caspase activation is inhibited by zVAD.fmk. However, our findings showing that the RIP1 inhibitor Nec-1 or RNAi-mediated silencing of RIP3 fail to protect from Erastin/BV6-mediated cell death do not support this hypothesis, as RIP1 and RIP3 are considered to be key elements of the necroptotic pathway [18].

We previously reported that pharmacological blockage of antioxidant pathways responsible for the detoxification of ROS, using buthionine sulfoximine (BSO), a specific inhibitor of the rate-limiting enzyme in GSH synthesis γ-glutamylcysteine ligase [21], Auranofin, an inhibitor of thioredoxin reductase, a key enzyme in the antioxidant thioredoxin pathway [3], or Erastin can prime ALL cells for Smac mimetic-induced cell death [16, 22]. These findings underscore the relevance of redox signaling in the regulation of Smac mimetic-mediated cell death.

Taken together, RSL3/BV6 and Erastin/BV6 differentially regulate redox signaling and cell death in ALL cells. While RSL3/BV6 cotreatment induces ferroptotic cell death, Erastin/BV6 cotreatment stimulates oxidative cell death independently of iron. These findings have important implications for the therapeutic targeting of redox signaling to reactivate programmed cell death in ALL.

## MATERIALS AND METHODS

### Cell culture and chemicals

ALL cell lines were obtained from DSMZ (Braunschweig, Germany) and cultured in RPMI 1640 or Dulbecco's Modified Eagle Medium (DMEM) medium (Life Technologies, Inc., Eggenstein, Germany), supplemented with 10% FCS (fetal calf serum) (Biochrom, Berlin, Germany), 1% penicillin/streptomycin (Invitrogen) and 25 mM HEPES (Biochrom). The bivalent Smac mimetic BV6, which antagonizes XIAP, cIAP1 and cIAP2 [14], was kindly provided by Genentech Inc. (South San Francisco, CA, USA). Erastin, Fer-1, DFO and α-Toc were purchased from Sigma-Aldrich (Taufkirchen, Germany), zVAD.fmk from Bachem (Heidelberg, Germany), Nec-1 from Merck (Darmstadt, Germany) and recombinant human TNFα from Biochrom (Berlin, Germany). RSL3 was kindly provided by B. Stockwell (Columbia University, New York, NY, USA) or purchased from InterBIOScreen Ltd. (Moscow, Russia). All chemicals were purchased by Sigma-Aldrich or Carl Roth (Karlsruhe, Germany) unless indicated otherwise.

### Gene silencing and transduction

Transient gene silencing by small interfering RNA (siRNA) was performed as previously described [23] using Neon Transfection System (Invitrogen) and Silencer^®^ Select siRNAs against RIP3 (s21741) or non-targeting control siRNA (s4390843). Human GPX4 was stably overexpressed by lentiviral transduction. Shortly, packaging Phoenix cells were transfected with 20 μg pMSCV plasmid (empty vector, GPX4) using calcium phosphate transfection. Virus-containing supernatant was collected, sterile-filtered and used for spin transduction at 37°C in the presence of 4 μg/ml protamine sulfate. Transduced Molt-4 cells were selected by 1 μg/ml pyromycin (Roth).

### Determination of cell death, ROS production and lipid peroxidation

Cell death was assessed by forward/side scatter (FSC/SSC) analysis and flow cytometry (FACSCanto II, BD Biosciences, Heidelberg, Germany) as described previously [24]. ROS production and lipid peroxidation were analyzed in PI-negative cells before cells succumb to cell death as previously described [16]. To analyze ROS production, cells were incubated with 1 μM CellROX (Invitrogen) for 30 minutes at 37°C and immediately analyzed by flow cytometry. Lipid peroxidation was analyzed by staining cells with 5 μM BODIPY-C11 (Invitrogen) for 30 minutes at 37°C followed by flow cytometric analysis.

### Western blot analysis

Western blot analysis was performed as described previously [25] using the following antibodies: mouse anti-GPX4 (R&D Systems, Inc., Wiesbaden, Germany), rabbit anti-RIP3 (Imgenex, San Diego, CA, USA), mouse anti-β-Actin (Sigma-Aldrich) or mouse anti-GAPDH (HyTest, Turku, Finland). Goat anti-mouse IgG or goat anti-rabbit IgG conjugated to horseradish peroxidase (Santa Cruz Biotechnology, Santa Cruz, CA) and enhanced chemiluminescence (Amersham Bioscience, Freiburg, Germany) or infrared dye-labeled secondary antibodies and infrared imaging (Odyssey Imaging System, LI-COR Bioscience, Bad Homburg, Germany) were used for detection. Representative blots of at least two independent experiments are shown.

### Determination of caspase-3/7 activity

Caspase-3/7 activity was detected by Cell Event Caspase-3/7 Green Detection Reagent (Life Technologies, Inc., Eggenstein, Germany) according to the manufacturer's instructions using ImageXpress Micro XLS system (Molecular Devices, Biberach an der Riss, Germany).

### Statistical analysis

Statistical significance was assessed by Student's *t*-test (two-tailed distribution, two-sample, unequal variance) using Microsoft Excel (Microsoft Deutschland GmbH, Unterschleißheim, Germany).

## SUPPLEMENTARY MATERIALS FIGURES


