# Supplementary material for: RSL3 and Erastin differentially regulate redox signaling to promote Smac mimetic-induced cell death
Source: Oncotarget. 2016 Aug 29;7(39):63779–92. doi: 10.18632/oncotarget.11687 (PMC5325403; doi:10.18632/oncotarget.11687)
Supplement: Supplementary file 1 [file oncotarget-07-63779-s001.pdf]

RSL3 and Erastin differentially regulate redox signaling to promote Smac mimetic-induced cell death

Supplementary Materials

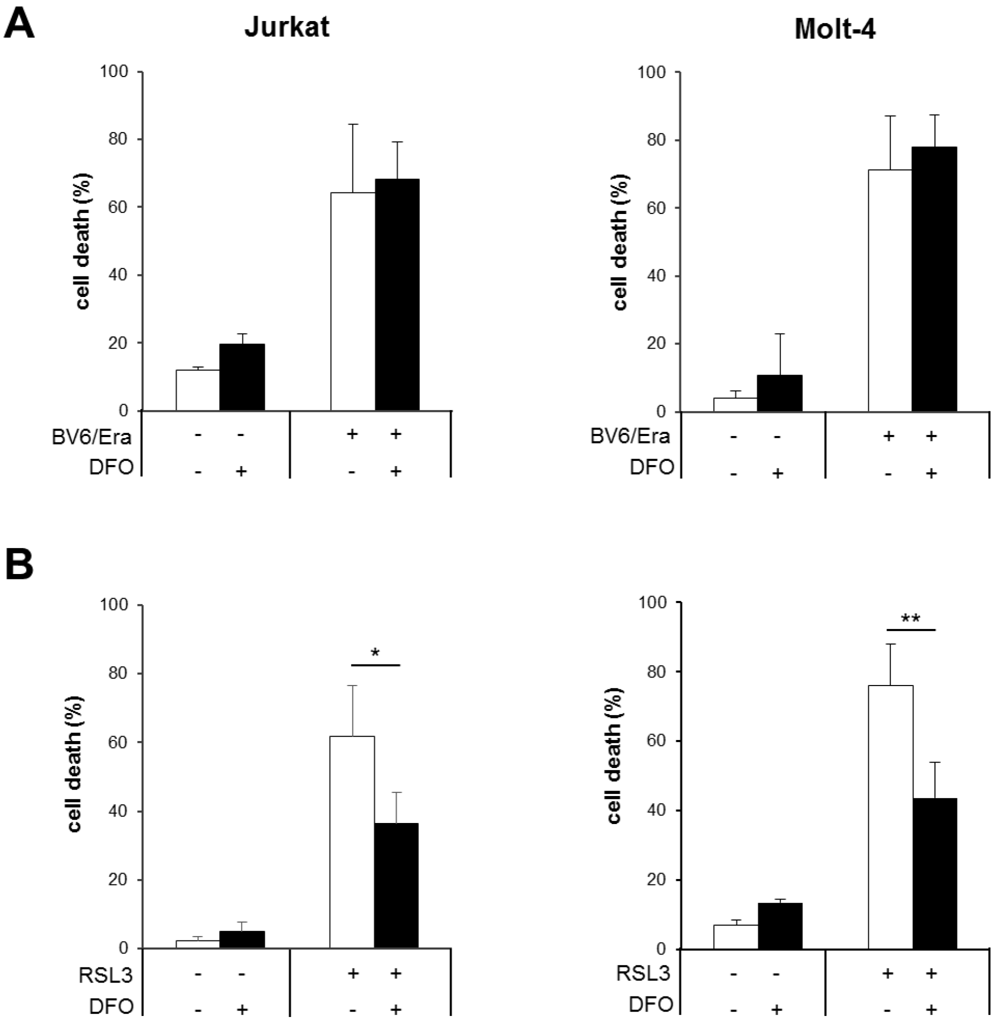

Supplementary Figure S1: RSL3/BV6 but not Erastin/BV6 cotreatment triggers irondependent cell death. (**A**) ALL cells were treated for 24 hours with BV6 (Jurkat: 5 µM, Molt-4: 4 µM) and Erastin (Era) (Jurkat: 5 µM; Molt-4: 7.5 µM) in the presence or absence of 50 µM DFO, which was added 2 hours before treatment. Cell death was determined by FSC/SSC analysis and flow cytometry. Mean and SD of at least three experiments performed in triplicate are shown. (**B**) ALL cells were treated for 24 hours with RSL3 (Jurkat: 10 µM, Molt-4: 0.2 µM) in the presence or absence of 25 µM DFO, which was added 2 hours before treatment. Cell death was determined by FSC/SSC analysis and flow cytometry. Mean and SD of at least three experiments performed in triplicate are shown; $*P < 0.05$; $**P < 0.01$.

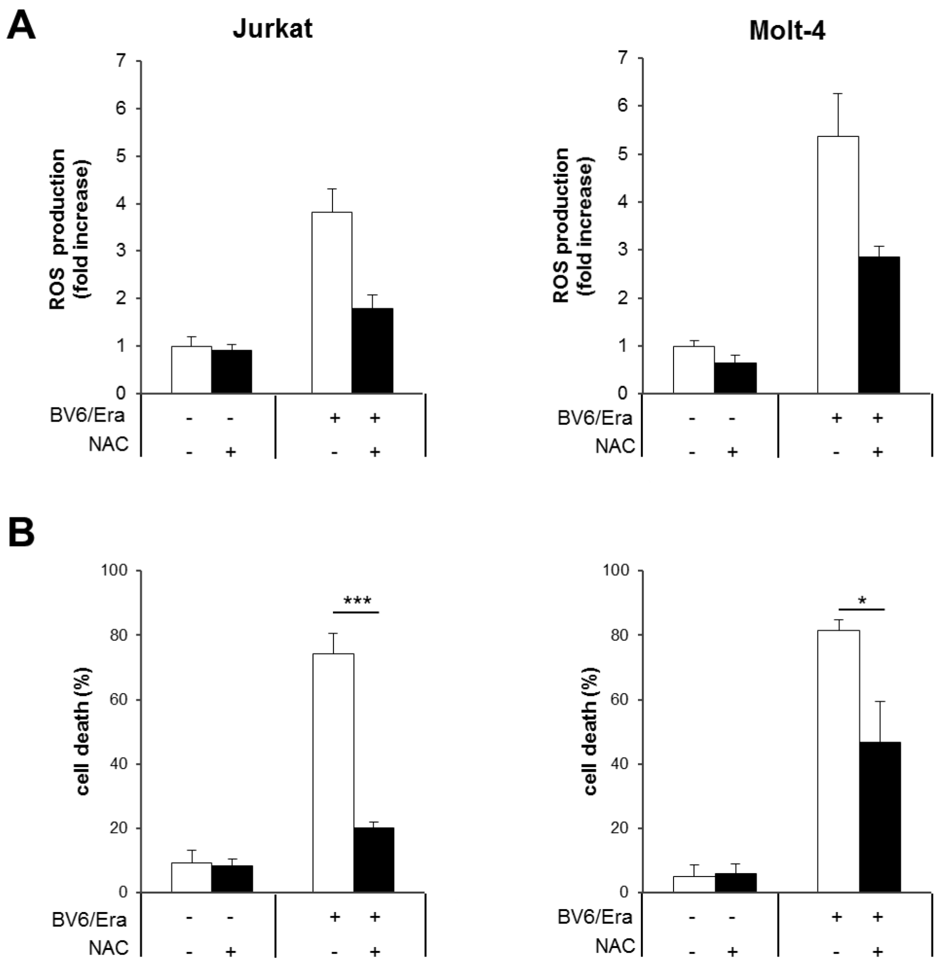

Supplementary Figure S2: NAC protects from Erastin/BV6-induced cell death. ALL cells were treated with BV6 (Jurkat: 5 µM, Molt-4: 4 µM) and Erastin (Era) (Jurkat: 5 µM; Molt-4: 7.5 µM) in the presence or absence of 10 mM NAC, which was added 2 hours before treatment. ROS production was determined after 15 hours by flow cytometry in PI-negative cells using the fluorescent dye CellROX and is shown as fold increase (**A**). Cell death was determined after 24 hours by FSC/SSC analysis and flow cytometry (**B**). Mean and SD of one (A) or three (B) experiments performed in triplicate are shown; *$P < 0.05$; ***$P < 0.001$.

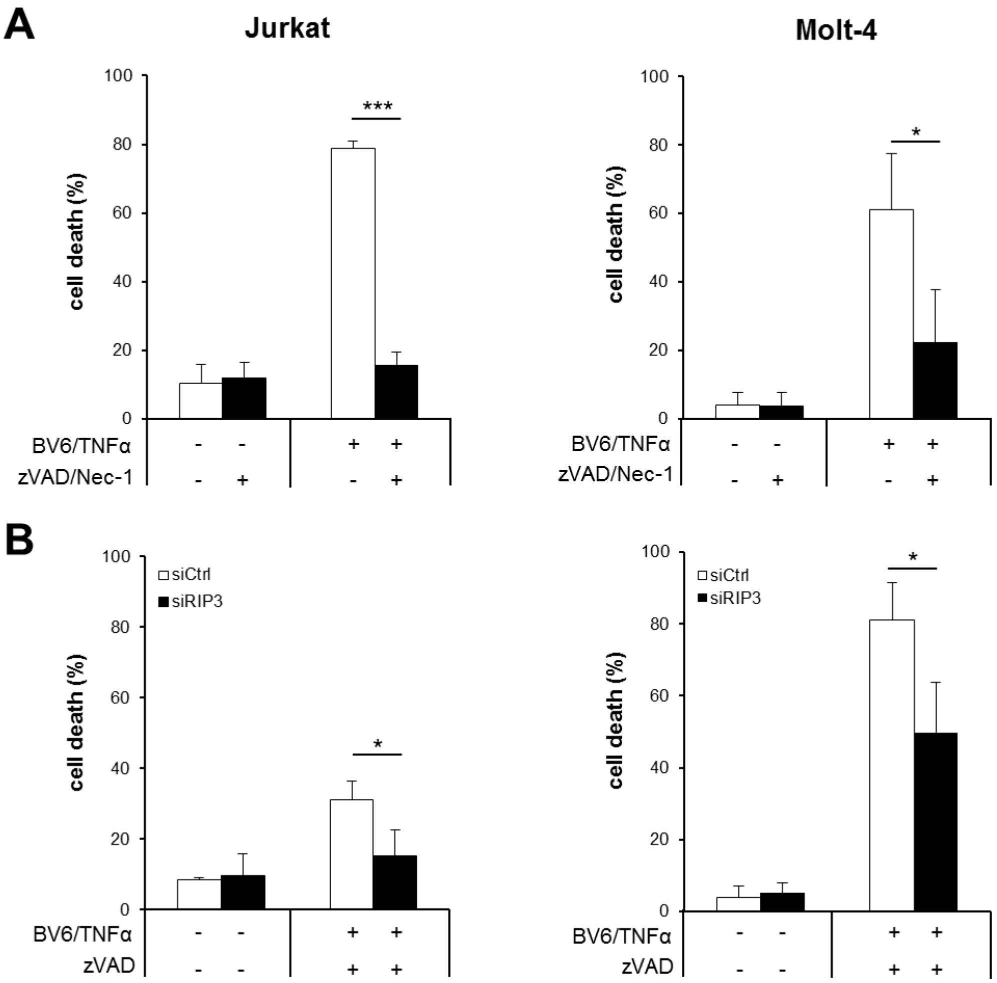

Supplementary Figure S3: TNFα/BV6 cotreatment triggers RIP1- and RIP3-dependent cell death. (**A**) ALL cells were treated with TNFα (Jurkat: 1 ng/ml, Molt-4: 100 ng/ml) and BV6 (Jurkat: 1 µM, Molt-4: 4 µM) in presence or absence of 20 µM zVAD.fmk and 15 µM Nec-1, which were added 2 hours before treatment. Cell death was determined after 24 hours by FSC/SSC analysis and flow cytometry. (**B**) ALL cells were transiently transfected with siRNA against RIP3 or control siRNA and cell death was determined after 24 hour treatment TNFα (Jurkat: 1 ng/ml, Molt-4: 100 ng/ml) and BV6 (Jurkat: 1 µM, Molt-4: 4 µM) in the presence or absence of 20 µM zVAD.fmk by FSC/SSC analysis and flow cytometry. Mean and SD of three experiments performed in triplicate are shown; *$P < 0.05$; ***$P < 0.001$.

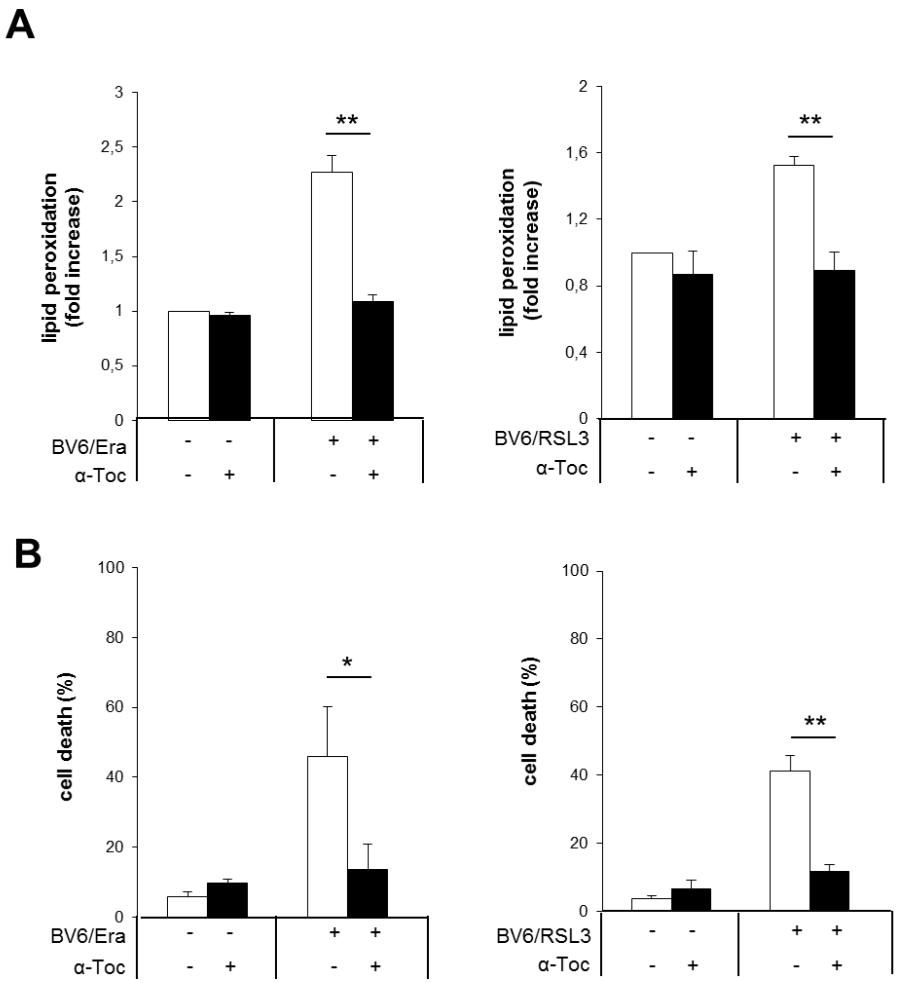

Supplementary Figure S4: α-Toc inhibits RSL3/BV6- or Erastin/BV6-induced lipid peroxidation and cell death. (**A**) Reh cells were treated with 0.3 µM BV6 and 15 µM Erastin (Era) or with 0.3 µM BV6 and 0.3 µM RSL3 in the presence or absence of 100 µM α-Toc. Lipid peroxidation was assessed after 48 hours by flow cytometry in PI-negative cells using the fluorescent dye BODIPY-C11 and is shown as fold increase (A). Cell death was determined after 48 hours (BV6/Erastin) or 72 hours (BV6/RSL3) by FSC/SSC analysis and flow cytometry (**B**). Mean and SD of three experiments performed in triplicate are shown; *$P < 0.05$; **$P < 0.01$.

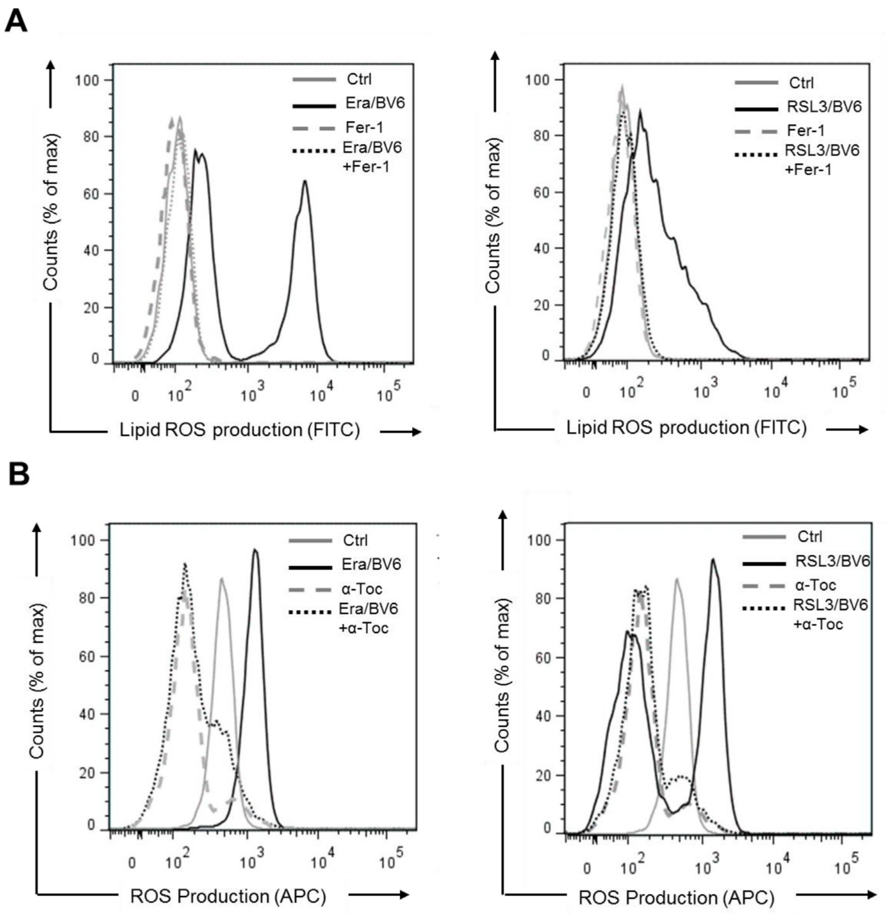

Supplementary Figure S5: Effect of Fer-1 or α-Toc on RSL3/BV6- or Erastin/BV6-induced lipid peroxidation or ROS production. (**A**) Jurkat cells were treated with 5 μM BV6 and 5 μM Erastin (Era) or 5 μM BV6 and 0.1 μM RSL3 in the presence or absence of 5 μM Fer-1, which was added 2 hours before 3 treatment. Lipid peroxidation was assessed after 12 hours (Erastin/BV6) or 24 hours (RSL3/BV6) by flow cytometry in PI-negative cells using the fluorescent dye BODIPY-C11 and is shown as fold increase. (**B**) Jurkat cells were treated with 5 μM BV6 and 5 μM Erastin or 5 μM BV6 and 0.1 μM RSL3 in the presence or absence of 100 μM α-Toc, which were added 2 hours before treatment. ROS production was determined after 15 hours by flow cytometry in PI-negative cells using the fluorescent dye CellROX.
